# Overview of miRNAs for the non-invasive diagnosis of endometriosis: evidence, challenges and strategies. A systematic review

**DOI:** 10.31744/einstein_journal/2021RW5704

**Published:** 2021-04-15

**Authors:** Vitor Ulisses Monnaka, Camila Hernandes, Debora Heller, Sérgio Podgaec

**Affiliations:** 1 Faculdade Israelita de Ciências da Saúde Albert Einstein Hospital Israelita Albert Einstein São PauloSP Brazil Faculdade Israelita de Ciências da Saúde Albert Einstein, Hospital Israelita Albert Einstein, São Paulo, SP, Brazil.; 2 Hospital Israelita Albert Einstein São PauloSP Brazil Hospital Israelita Albert Einstein, São Paulo, SP, Brazil.

**Keywords:** Biomarkers, Saliva, Serum, Vaginal fluid, Body fluids banks, MicroRNAs, Endometriosis/diagnosis

## Abstract

**Objective:**

The aim of the study was to assess the evidence on miRNAs as biomarkers for the diagnosis of endometriosis, as well as to provide insights into the challenges and strategies associated with the use of these molecules as accessible tools in clinical practice.

**Methods:**

Systematic review conducted on PubMed^®^, Latin American and Caribbean Health Sciences Literature (LILACS), MEDLINE^®^ and Web of Science databases using the search terms endometriosis (all fields) AND miRNA (all fields), evaluating all publication up to May 2019.

**Results:**

Most miRNAs found to be dysregulated in this study were harvested from tissue samples, which precludes their use as a non-invasive diagnostic test. However, differential expression of 62 miRNAs was reported in samples that may be used for non-invasive diagnosis of endometriosis, such as blood, serum and plasma.

**Conclusion:**

Despite the identification of several candidates, studies are investigatory in nature and have been conducted with small number of samples. Also, no particular miRNA has been validated for diagnostic purposes so far. Studies based primarily on biological samples and applicable to translational research are warranted. Large databases comprising information on sample type and the use of saliva and vaginal fluid for miRNAs identification may prove essential to overcome current barriers to diagnosis of endometriosis.

## INTRODUCTION

Endometriosis is a common disease that affects up to 10% of women of reproductive age^(1,2)^ and is characterized by the presence of endometrial cells outside the uterine cavity. The disease has been the focus of many studies, however, the diagnosis is still very difficult. Clinical presentation varies widely, ranging from asymptomatic to severe, and no diagnostic biomarkers have been approved for routine clinical diagnosis of endometriosis to date.^(1,3)^

Diagnostic imaging tests such as pelvic ultrasonography and magnetic resonance have been used, especially in deep endometriosis. However, examiner expertise has a strong impact on imaging findings,^(4-7)^ which ultimately makes the diagnosis difficult. In cases with no positive imaging findings, a final diagnosis of superficial endometriosis can only be made through histological analysis of the lesion, usually in samples obtained by laparoscopic surgery.^(8,9)^ However, this procedure is invasive and requires general anesthesia.

The complexity of the disease, combined with the lack of precise and less invasive diagnostic methods, contributes to delayed diagnosis, which can take up to 11 years.^(5,10,11)^ Therefore, there is great demand for accurate and less invasive diagnostic tests for endometriosis.^(12-16)^

Different research groups have investigated the role of miRNAs (microRNAs or miR) in the regulation of known genes, given their association with processes involved in disease pathogenesis and progression. miRNAs are a class of small endogenous, non-coding RNA molecules involved in post-transcriptional regulation of gene expression.^(17)^ These small molecules have also been found in peripheral blood and may therefore be potential diagnostic biomarkers for endometriosis.^(18,19)^

This literature search was conducted to determine how close miRNAs are to being used as biomarkers for endometriosis. Findings of this review are expected to guide the next steps towards overcoming challenges associated with the use of miRNAs in clinical practice.

## OBJECTIVE

To determine which miRNAs are applicable to the diagnosis of endometriosis and to outline the challenges and strategies involved in the use of these molecules as accessible diagnostic tools in clinical settings.

## METHODS

To identify research articles addressing associations between endometriosis and miRNA, a search was conducted in PubMed^®^, Latin American and Caribbean Health Sciences Literature (LILACS), MEDLINE^®^ and Web of Science databases using the search terms endometriosis (all fields) AND miRNA (all fields).

All publications listed up to May 2019 (automatically selected) were manually curated, and only those involving miRNA expression patterns, validated by polymerase chain reaction (PCR) in clinical samples of endometriosis, were discussed in this review. Articles published in languages other than English or based on cell culture, retracted articles and articles published in conference proceedings or inaccessible were excluded. Reports listed in more than one database were included only once in the pool of publications.

This study was conducted according to the Preferred Reporting Items for Systematic Reviews and Meta-Analyses (PRISMA) statement for systematic reviews adopted by *Hospital Israelita Albert Einstein* (HIAE), located in São Paulo (SP), Brazil. Data were extracted in duplicate and independently by two different investigators, then compared for confirmation. miRNAs and their respective expression levels in different types of samples and patient populations were examined. Studies were also analyzed according to year and country of publication.

## RESULTS

### Overview of publications on miRNA and endometriosis

**Figure 1:**
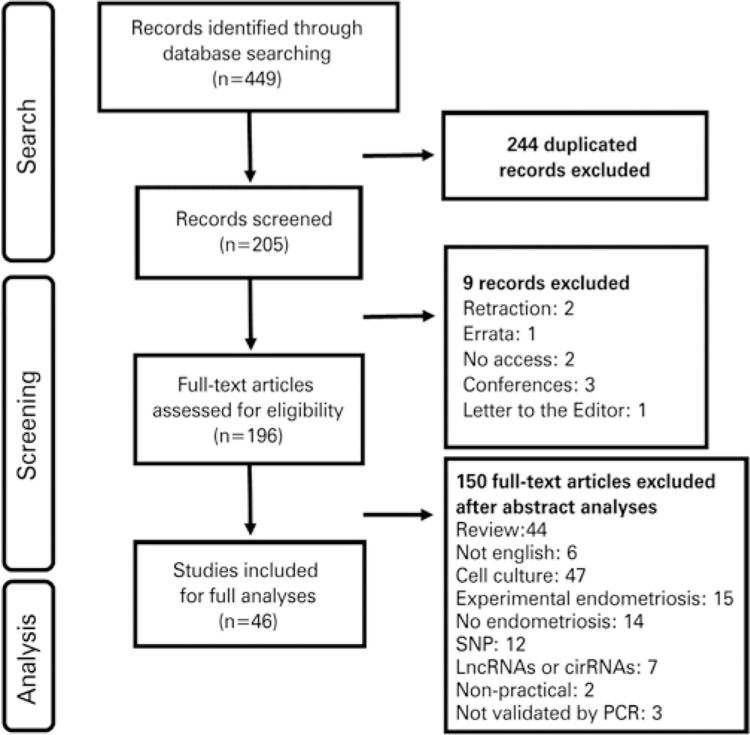
Summarized results of database screening for publications addressing associations between endometriosis and miRNAs SNP: single-nucleotide polymorphisms; LncRNAs: long non-coding ribonucleic acids; circRNAs: circular ribonucleic acids; PCR: polymerase chain reaction.

A total of 449 research articles addressing associations between endometriosis and miRNA were found in databases selected for this review. Most (185) were retrieved from PubMed^®^, followed by LILACS and MEDLINE^®^ (158) and Web of Science (106). Out of this publication pool, 46 matched final selection criteria and were selected for further discussion in this review (Figure 1).

The number of publications investigating dysregulated miRNAs in women with endometriosis increased sharply since 2009, with approximately half of selected articles (23) published in the last 3 years. China and the United States were the countries with the largest number of publications (21 and 9 articles, respectively).

Within this pool of 46 studies, 43 investigated miRNAs found to be dysregulated in the ectopic (EC) relative to the eutopic endometrium of control patients (EN), 25 were detected in the EC relative to the eutopic endometrium (EU) of women with endometriosis and 23 were detected in the EU compared to the EN group. Furthermore, 27 were detected in the serum, 18 in the plasma, 30 in the blood and six in the peritoneal fluid of women with endometriosis compared to the Control Group. Blood seems to be the most widely investigated type of sample regarding potential applicability to non-invasive diagnosis. The summary of dysregulated miRNA found in selected published articles are listed in table 1A-1G [Table t2] [Table t3] [Table t4] [Table t5] [Table t6] [Table t7].

**Table 1A:** miRNAs differentially expressed in eutopic endometrium of endometriosis patients compared with eutopic endometrium of control patients

miRNA	Regulation	Endometriosis n	Control n	References
miR-126	DR	31	27	Liu et al.^(20)^
miR-1281	UR	38	38	Yang et al.^(21)^
miR-142-5p	UR	38	38	Yang et al.^(21)^
miR-145	UR	11	22	Zheng et al.^(22)^
miR-146a-5p	UR	38	38	Yang et al.^(21)^
miR-183-5p	DR	N/A	N/A	Shi et al.^(23)^
miR-199a	DR	12	12	Dai et al.^(24)^
miR-202-3p	DR	51	32	Braza-Boïls et al.^(25)^
miR-204	DR	38	9	Haikalis et al.^(26)^
miR-29c	DR	20	10	Long et al.^(27)^
miR-30d-5p	UR	21	25	Laudanski et al.^(28)^
miR-3152-5p	UR	21	25	Laudanski et al.^(28)^
miR-34b	DR	4	3	Burney et al.^(29)^
miR-34c-5p	DR	4	3	Burney et al.^(29)^
miR-424-5p	DR	51	32	Braza-Boils et al.^(25)^
miR-4634	UR	38	38	Yang et al.^(21)^
miR-483-5p	DR	21	25	Laudanski et al.^(30)^
miR-5187-3p	UR	21	25	Laudansk et al.^(28)^
miR-543	DR	38	38	Yang et al.^(21)^
miR-556-3p	DR	51	32	Braza-Boïls et al.^(25)^
miR-629*	DR	21	25	Laudanski et al.^(30)^
miR-9	DR	4	3	Burney et al.^(29)^
miR-940	UR	38	38	Yang et al.^(21)^

miRNA: microRNA; DR: downregulated; UR: upregulated; N/A: not available.

**Table 1B t2:** miRNAs differentially expressed in ectopic endometrium of endometriosis patients and eutopic endometrium of control patients

miRNA	Regulation	Endometriosis n	Control n	References
let-7g	UR	N/A	N/A	Wright et al.^(31)^
miR-100	UR	N/A	N/A	Wright et al.^(31)^
miR-1304-3p	UR	14	10	Xu et al.^(32)^
miR-133a-3p	UR	33	17	Braicu et al.^(33)^
miR-138	UR	51	32	Braza-Boïls et al.^(25)^
miR-141	UR	22	24	Saare et al.^(34)^
miR-143	UR	11	22	Zheng et al.^(22)^
miR-145	UR	11	22	Zheng et al.^(22)^
miR-148a	UR	N/A	N/A	Wright et al.^(31)^
miR-183-5p	DR	N/A	N/A	Shi et al.^(23)^
miR-191	UR	12	12	Dong et al.^(35)^
miR-199a	DR	12	12	Dai et al.^(24)^
miR-200a	UR	22	24	Saare et al.^(34)^
miR-200b	UR	22	24	Saare et al.^(34)^
miR-200c	DR	27	12	Liang et al.^(36)^
miR-202-3p	UR	51	32	Braza-Boïls et al.^(25)^
miR-205-5p	DR	14	10	Xu et al.^(32)^
miR-20a	UR	40	20	Zhao et al.^(37)^
miR-21-3p	UR	7	7	Qi et al.^(38)^
miR-223-3p	UR	7	7	Qi et al.^(38)^
miR-29a	UR	N/A	N/A	Wright et al.^(31)^
miR-29c	DR	20	10	Long et al.^(27)^
miR-29c	UR	51	32	Braza-Boïls et al.^(25)^
miR-29c	UR	15	11	Joshi et al.^(39)^
miR-325	UR	33	17	Braicu et al.^(33)^
miR-33b	DR	20	15	Yang et al.^(40)^
miR-34c	UR	22	24	Saare et al.^(34)^
miR-3663-3p	UR	7	7	Qi et al.^(38)^
miR-3684	UR	14	10	Xu et al.^(32)^
miR-373-3p	UR	51	32	Braza-Boïls et al.^(25)^
miR-3935	DR	14	10	Xu et al.^(32)^
miR-411-5p	UR	51	32	Braza-Boïls et al.^(25)^
miR-4427	DR	14	10	Xu et al.^(32)^
miR-449a	UR	22	24	Saare et al.^(34)^
miR-450a-5p	DR	7	7	Qi et al.^(38)^
miR-451	UR	30	0	Graham et al.^(41)^
miR-4683	UR	14	10	Xu et al.^(32)^
miR-492	UR	33	17	Braicu et al.^(33)^
miR-494-5p	UR	14	10	Xu et al.^(32)^
miR-503-5p	DR	7	7	Qi et al.^(38)^
miR-520e	UR	33	17	Braicu et al.^(33)^
miR-544b	UR	14	10	Xu et al.^(32)^
miR-5481	DR	N/A	N/A	Wright et al.^(31)^
miR-652-5p	DR	14	10	Xu et al.^(32)^
miR-6747-3p	UR	14	10	Xu et al.^(32)^

miRNA: microRNA; DR: downregulated; N/A: not available; UR: upregulated.

**Table 1C t3:** miRNAs differentially expressed in the ectopic and eutopic endometrium of endometriosis patients

miRNA	Regulation	Endometriosis n	Control n	References
miR-106a-5p	DR	22	0	Zhao et al.^(42)^
miR-106b-5p	UR	32	19	Yang et al.^(43)^
miR-10a	DR	38	38	Haikalis et al.^(26)^
miR-125a	UR	58	38	Ramón et al.^(44)^
miR-126	DR	31	27	Liu et al.^(20)^
miR-126	UR	8	N/A	Ohlsson Teague et al.^(45)^
miR-141	DR	8	N/A	Ohlsson Teague et al.^(45)^
miR-145	UR	8	N/A	Ohlsson Teague et al.^(45)^
miR-145-5p	UR	32	19	Yang et al.^(43)^
miR-146a-5p	DR	32	19	Yang et al.^(43)^
miR-15a-5p	DR	32	19	Yang et al.^(43)^
miR-16-5p	UR	32	19	Yang et al.^(43)^
miR-182	DR	16	N/A	Filigheddu et al.^(46)^
miR-182-5p	DR	22	0	Zhao et al.^(42)^
miR-19b-1-5p	DR	32	19	Yang et al.^(43)^
miR-200a	DR	16	N/A	Filigheddu et al.^(46)^
miR-200a-3p	DR	22	0	Zhao et al.^(42)^
miR-200b	DR	16	N/A	Filigheddu et al.^(46)^
miR-200b	DR	8	N/A	Ohlsson Teague et al.^(45)^
miR-200b	DR	32	19	Yang et al.^(43)^
miR-200c	DR	16	N/A	Filigheddu et al.^(46)^
miR-200c	DR	32	19	Yang et al.^(43)^
miR-202	UR	16	N/A	Filigheddu et al.^(46)^
miR-21	DR	38	38	Haikalis et al.^(26)^
miR-222	UR	58	38	Ramón et al.^(44)^
miR-34c	DR	22	0	Zhao et al.^(42)^
miR-424	DR	8	N/A	Ohlsson Teague et al.^(45)^
miR-424	DR	38	38	Haikalis et al.^(26)^
miR-449b	DR	51	32	Braza-Boïls et al.^(25)^
miR-449b	DR	22	0	Zhao et al.^(42)^
miR-451a	UR	41	40	Nothnick et al.^(47)^
miR-615	UR	22	0	Zhao et al.^(42)^
miR-9	DR	38	38	Haikalis et al.^(26)^
miR-99a	UR	8	N/A	Ohlsson Teague et al.^(45)^

miRNA: microRNA; DR: downregulated; UR: upregulated; N/A: not available.

**Table 1D t4:** miRNAs differentially expressed in the serum of endometriosis and control patients

miRNA	Regulation	Endometriosis n	Control n	References
let-7b	DR	24	24	Cho et al.^(48)^
let-7b-5p	DR	20	26	Nematian et al.^(49)^
miR-122	UR	60	25	Wang et al.^(19)^
miR-122	UR	45	35	Maged et al.^(50)^
miR-125b-5p	UR	24	24	Cosar et al.^(51)^
miR-125b	UR	20	26	Nematian et al.^(49)^
miR-127-3p	DR	30	20	Wang et al.^(52)^
miR-135a	DR	24	24	Cosar et al.^(51)^
miR-141	DR	60	25	Wang et al.^(19)^
miR-143-3p	UR	24	24	Cosar et al.^(51)^
miR-145	DR	60	25	Wang et al.^(19)^
miR-145-5p	UR	24	24	Cosar et al.^(51)^
miR-150-5p	UR	24	24	Cosar et al.^(51)^
miR-15b-5p	DR	30	20	Wang et al.^(52)^
miR-17	DR	80	60	Wang et al.^(53)^
miR-185-5p	UR	30	20	Wang et al.^(52)^
miR-18a-5p	UR	24	24	Cosar et al.^(51)^
miR-191	UR	12	12	Dong et al.^(35)^
miR-199a	UR	60	25	Wang et al.^(19)^
miR-199a	UR	45	35	Maged et al.^(50)^
miR-199a-5p	DR	40	25	Hsu et al.^(54)^
miR-20a-5p	DR	30	20	Wang et al.^(52)^
miR-30c-5p	DR	30	20	Wang et al.^(52)^
miR-342-3p	UR	24	24	Cosar et al.^(51)^
miR-3613-5p	DR	24	24	Cosar et al.^(51)^
miR-370	DR	20	26	Hu et al.^(55)^
miR-424-3p	UR	30	20	Wang et al.^(52)^
miR-451a	UR	41	40	Nothnick et al.^(47)^
miR-451a	UR	24	24	Cosar et al.^(51)^
miR-500a-3p	UR	24	24	Cosar et al.^(51)^
miR-542-3p	DR	60	25	Wang et al.^(19)^
miR-6755-3p	DR	24	24	Cosar et al.^(51)^
miR-9	DR	60	25	Wang et al.^(19)^
miR-99b-5p	DR	30	20	Wang et al.^(52)^

miRNA: microRNA; DR: downregulated; UR: upregulated.

**Table 1E t5:** miRNAs differentially expressed in the plasma of endometriosis and control patients

miRNA	Regulation	Endometriosis n	Control n	References
miR-139	DR	80	39	Nisenblat et al.^(56)^
miR-141	DR	61	65	Rekker et al.^(57)^
miR-145	UR	55	23	Bashti et al.^(58)^
miR-154-5p	DR	51	41	Pateisky et al.^(59)^
miR-155	DR	80	39	Nisenblat et al.^(56)^
miR-16	UR	33	20	Suryawanshi et al.^(60)^
miR-17-5p	DR	23	23	Jia et al.^(61)^
miR-191	UR	33	20	Suryawanshi et al.^(60)^
miR-195	UR	33	20	Suryawanshi et al.^(60)^
miR-196b	DR	51	41	Pateisky et al.^(59)^
miR-200a	DR	61	65	Rekker et al.^(57)^
miR-200b	DR	61	65	Rekker et al.^(57)^
miR-20a	DR	23	23	Jia et al.^(61)^
miR-22	DR	23	23	Jia et al.^(61)^
miR-31	DR	55	23	Bashti et al.^(58)^
miR-33a	UR	51	41	Pateisky et al.^(59)^
miR-378a	DR	51	41	Pateisky et al.^(59)^
miR-574	DR	80	39	Nisenblat et al.^(56)^

miRNA: microRNA; DR: downregulated; UR: upregulated.

**Table 1F t6:** miRNAs differentially expressed in the blood of endometriosis and control patients

miRNA	Regulation	Endometriosis n	Control n	References
let-3c	DR	4	3	Azmy et al.^(62)^
let-7e	DR	4	3	Azmy et al.^(62)^
let-7f	DR	5	3	Azmy et al.^(62)^
let-7g	DR	4	3	Azmy et al.^(62)^
miR-103	DR	4	3	Azmy et al.^(62)^
miR-106b	DR	4	3	Azmy et al.^(62)^
miR-125a-5p	DR	4	3	Azmy et al.^(62)^
miR-126	DR	4	3	Azmy et al.^(62)^
miR-15b	DR	4	3	Azmy et al.^(62)^
miR-16	DR	4	3	Azmy et al.^(62)^
miR-17	DR	4	3	Azmy et al.^(62)^
miR-181b	DR	4	3	Azmy et al.^(62)^
miR-18a	DR	4	3	Azmy et al.^(62)^
miR-194	DR	4	3	Azmy et al.^(62)^
miR-195	DR	4	3	Azmy et al.^(62)^
miR-19a	DR	4	3	Azmy et al.^(62)^
miR-19b	DR	4	3	Azmy et al.^(62)^
miR-20a	DR	4	3	Azmy et al.^(62)^
miR-21	DR	4	3	Azmy et al.^(62)^
miR-22	DR	4	3	Azmy et al.^(62)^
miR-26a	DR	4	3	Azmy et al.^(62)^
miR-26b	DR	4	3	Azmy et al.^(62)^
miR-27a	DR	4	3	Azmy et al.^(62)^
miR-27b	DR	4	3	Azmy et al.^(62)^
miR-30a	DR	4	3	Azmy et al.^(62)^
miR-374a	DR	4	3	Azmy et al.^(62)^
miR-374b	DR	4	3	Azmy et al.^(62)^
miR-424	DR	4	3	Azmy et al.^(62)^
miR-7	DR	4	3	Azmy et al.^(62)^
miR-93	DR	4	3	Azmy et al.^(62)^

miRNA: microRNA; DR: downregulated.

**Table 1G t7:** miRNAs differentially expressed in the peritoneal fluid of endometriosis and control patients

miRNA	Regulation	Endometriosis n	Control n	References
miR-106b-3p	UR	126	45	Marí-Alexandre et al.^(63)^
miR-122	UR	45	35	Maged et al.^(50)^
miR-130b	UR	6	3	Chen et al.^(64)^
miR-199a	UR	45	35	Maged et al.^(50)^
miR-451a	UR	126	45	Marí-Alexandre et al.^(63)^
miR-486-5p	UR	126	45	Marí-Alexandre et al.^(63)^

miRNA: microRNA; UR: upregulated.

A total of 33 miRNAs were examined in more than one study. Of these, 13 miRNAs were analyzed in the same types of samples. miRNAs identified in more than one study and body fluid are described in table 2.

**Table 2:** Summary of miRNA dysregulated identified in more than one study in different samples

Total	EU *versus* EN	EC *versus* EN	EC *versus* EU	Plasma	Serum	Blood	PF	References
6	miR-145	miR-145	miR-145	miR-145	miR-145			Wang et al.,^(19)^ Zheng et al.,^(22)^ Yang et al.,^(43)^ Ohlsson Teague et al.,^(45)^ Cosar et al.^(51)^ and Bashti et al.^(58)^
5		miR-200b	miR-200b	miR-200b				Saare et al.,^(34)^ Yang et al.,^(43)^ Ohlsson Teague et al.,^(45)^ Filigheddu et al.^(46)^ and Rekker et al.^(57)^
5	miR-424		miR-424		miR-424	miR-424		Braza-Boils et al.,^(25)^ Haikalis et al.,^(26)^ Ohlsson Teague et al.,^(45)^ Wang et al.^(52)^ and Azmy et al.^(62)^
4	miR-199a	miR-199a			miR-199a		miR-199a	Wang et al.,^(19)^ Dai et al.,^(24)^ Maged et al.^(50)^ and Hsu et al.^(54)^
4		miR-141	miR-141	miR-141	miR-141			Wang et al.,^(19)^ Saare et al.,^(34)^ Ohlsson Teague et al.^(45)^ and Rekker et al.^(57)^
4		miR-20a		miR-20a	miR-20a	miR-20a		Zhao et al.,^(37)^ Wang et al.,^(52)^ Jia et al.^(61)^ and Azmy et al.^(62)^
4		miR-200a	miR-200a	miR-200a				Saare et al.,^(34)^ Zhao et al.,^(42)^ Filigheddu et al.^(46)^ and Rekker et al.^(57)^
3	miR-29c	miR-29c	miR-29c					Braza-Boils et al.,^(25)^ Long et al.^(27)^ and Joshi et al.^(39)^
3	miR-34c	miR-34c	miR-34c					Braza-Boïls et al.,^(25)^ Saare et al.^(34)^ and Joshi et al.^(39)^
3		miR-200c	miR-200c					Liang et al.,^(36)^ Yang et al.^(43)^ and Filigheddu et al.^(46)^
3		miR-21	miR-21			miR-21		Haikalis et al.,^(26)^ Qi et al.^(38)^ and Azmy et al.^(62)^
3	miR-126		miR-126			miR-126		Liu et al.,^(20)^ Ohlsson Teague et al.^(45)^ and Azmy et al.^(62)^
3			miR-16	miR-16		miR-16		Yang et al.,^(43)^ Suryawanshi et al.^(60)^ and Azmy et al.^(62)^
3			miR-451a		miR-451a		miR-451a	Nothnick et al.,^(47)^ Cosar et al.^(51)^ and Marí-Alexandre et al.^(63)^
3	miR-9		miR-9		miR-9			Wang et al.,^(19)^ Haikalis et al.^(26)^ and Burney et al.^(29)^
3			miR-106b			miR-106b	miR-106b	Yang et al.,^(43)^ Azmy et al.^(62)^ a^nd^ Marí-Alexandre et al.^(63)^
3				miR-17	miR-17	miR-17		Wang et al.,^(53)^ Jia et al.^(61)^ and Azmy et al.^(62)^
2					miR-122		miR-122	Wang et al.^(19)^ and Maged et al.^(50)^
2			miR-449b					Braza-Boïls et al.^(25)^ and Zhao et al.^(42)^
2		miR-191		miR-191	miR-191			Dong et al.^(35)^ and Suryawanshi et al.^(60)^
2	miR-202	miR-202	miR-202					Braza-Boïls et al.^(25)^ and Filigheddu et al.^(46)^
2		miR-143			miR-143			Zheng et al.^(22)^ and Cosar et al.^(51)^
2				miR-22		miR-22		Jia et al.^(61)^ and Azmy et al.^(62)^
2		miR let-7g				miR let-7g		Wright et al.^(31)^ and Azmy et al.^(62)^
2					miR-15b	miR-15b		Wanget al.^(52)^ and Azmy et al.^(62)^
2			miR-125a			miR-125a		Ramón et al.^(44)^ and Azmy et al.^(62)^
2				miR-195		miR-195		Suryawanshi et al.^(60)^ and Azmy et al.^(62)^
2					miR-18a	miR-18a		Cosar et al.^(51)^ and Azmy et al.^(62)^
2			miR-19b			miR-19b		Yanget al.^(43)^ and Azmy et al.^(62)^
2	miR-146a		miR-146a					Yang et al.^(21)^ and Yang et al.^(43)^
2			miR-182					Zhao et al.^(42)^ and Filigheddu et al.^(46)^
2					miR-125b			Nematian et al.^(49)^ and Cosar et al.^(51)^
2					miR-let-7b			Cho et al.^(48)^ and Nematian et al.^(49)^

EU: eutopic endometrium of women with endometriosis; EN: eutopic endometrium of control patients; EC: ectopic endometrium; PF: peritoneal fluid.

Twenty out of 62 miRNAs identified in samples with potential applicability to minimally invasive diagnosis of endometriosis, such as blood, serum, and plasma, were also found to be dysregulated in other types of tissue, such as EC and eutopic endometrium, and in the peritoneal fluid. Of these, 35% were detected in the same type of tissue in more than one study, including miR-200b, miR-145, miR-199a, miR-424, miR-200a, miR-126, and miR-451a. Thirteen miRNAs were found to be up or downregulated, as follows: miR-125b, miR-let-7b, miR-122, miR-451a and miR-199a in serum; miR-29c in the EC relative to the EN Group; and miR-145, miR-200b, miR-424, miR-200a, miR-200c, miR-449b and miR-182 in the EC relative to the EC of women with endometriosis (Table 3).

**Table 3:** Characterization of miRNAs expression for upregulation and downregulation in different samples

miRNA	n	EU *versus* EN		EC *versus* EN		EC *versus* EU		Plasma	Serum	Blood	PF
		EU	EN	EC	EN	EC	EU				
miR-145	6	↑ (1)	↓ (1)	↑ (1)	↓ (1)	↑ (2)	↓ (2)	↑ (1)	↑ (1) /↓ (1)		
miR-200b	5			↑ (1)	↓ (1)	↓ (3)	↑ (3)	↓ (1)			
miR-424	5	↓ (1)	↑ (1)			↓ (2)	↑ (2)		↑ (1)	↓ (1)	
miR-199a	4	↓ (1)	↑ (1)	↓ (1)	↑ (1)			↑ (1)	↑ (2)/↓ (1)		↑ (1)
miR-141	4			↑ (1)	↓ (1)	↓ (1)	↑ (1)	↓ (1)	↓ (1)		
miR-20a	4			↑ (1)	↓ (1)			↓ (1)	↓ (1)	↓ (1)	
miR-200a	4			↑ (1)	↓ (1)	↓ (2)	↑ (2)	↓ (1)			
miR-29c	3	↓ (1)	↑ (1)	↑ (2) /↓ (1)	↓ (2) / ↑(1)	↓ (1)	↑ (1)				
miR-34c	3	↓ (1)	↑ (1)	↑ (1)	↓ (1)	↓ (1)	↑ (1)				
miR-200c	3			↓ (1)	↑ (1)	↓ (2)	↑ (2)				
miR-21	3			↑ (1)	↓ (1)	↓ (1)	↑ (1)			↓ (1)	
miR-126	3	↓ (1)	↑ (1)			↑ (1)/↓ (1)	↓ (1)/↑ (1)			↓ (1)	
miR-16	3					↑ (1)	↓ (1)	↑ (1)		↓ (1)	
miR-451a	3					↑ (1)	↓ (1)		↑ (2)		↑ (1)
miR-9	3	↓ (1)	↑ (1)			↓ (1)	↑ (1)		↓ (1)		
miR-106b	3					↑ (1)	↓ (1)			↓ (1)	↑ (1)
miR-17	3							↓ (1)	↓ (1)	↓ (1)	
miR-122	2								↑ (2)		↑ (1)
miR-449b	2					↓ (2)	↑ (2)				
miR-191	2			↑ (1)	↓ (1)			↑ (1)	↑ (1)		
miR-202	2	↓ (1)	↑ (1)	↑ (1)	↓ (1)	↑ (1)	↓ (1)				
miR-143	2			↑ (1)	↓ (1)				↑ (1)		
miR-22	2							↓ (1)		↓ (1)	
miR let-7g	2			↑ (1)	↓ (1)					↓ (1)	
miR-15b	2								↓ (1)	↓ (1)	
miR-125a	2					↑ (1)	↓ (1)			↓ (1)	
miR-195	2							↑ (1)		↓ (1)	
miR-18a	2								↑ (1)	↓ (1)	
miR-19b	2					↓ (1)	↑ (1)			↓ (1)	
miR-146a	2	↑ (1)	↓ (1)			↓ (1)	↑ (1)				
miR-182	2					↓ (2)	↑ (2)				
miR-125b	2								↑ (2)		
miR-let-7b	2								↓ (2)		

↑ upregulation; ↓ downregulation.

miRNA: microRNA; EU: eutopic endometrium of women with endometriosis; EN: eutopic endometrium of control patients; EC: ectopic endometrium; PF: peritoneal fluid.

## DISCUSSION

Endometriosis can be a debilitating disease and may lead to poor quality of life.^(65)^ The disease is associated with dysmenorrhea, deep dyspareunia, chronic pelvic pain and infertility^(66,67)^ and is considered a public health concern, given the impact on patient physical and psychological health, and the socioeconomic impact of diagnosis, treatment and clinical control costs.^(68)^

The final diagnosis of endometriosis is currently based on histological analysis of the lesion, usually in samples obtained by laparoscopic surgery.^(69)^ However, imaging modalities are important non-invasive diagnostic alternatives for ovarian and deep endometriosis. Both surgical and non-surgical approaches require considerable professional skill and availability of specific data, which may represent a huge economic and health burden in developing countries.^(4-9)^

In the last three decades, researchers worldwide have tried to identify a non-invasive test that could shorten the turnaround time for diagnosis of endometriosis. CA-125 can be detected in blood or peritoneal fluid and is one of the best studied biomarkers. In some case studies, measurement of CA-125 levels was deemed promising, especially for diagnosis of more invasive endometriosis, provided measurements are made in the beginning of the menstrual cycle.^(70-72)^

In spite of conflicting results regarding the value of CA-125 as a final and important biomarker reported in recent reviews, according to Socolov et al., CA-125 is still the most recommended biomarker for endometriosis diagnosis and monitoring.^(73)^ In a more recent Cochrane review published in 2016, Nisenblat et al. compared the accuracy of any combination of non-invasive diagnostic tests to surgical diagnosis of pelvic endometriosis, using randomized controlled trials or cross-sectional studies published until early 2015 as a reference standard. Authors concluded that none of the biomarkers investigated (including CA-125) could be duly evaluated due to insufficient or poor-quality evidence, given the high heterogeneity and risk of bias in selected studies.^(15)^

CA-125 is most elevated in advanced stages of endometriosis. Therefore, the sensitivity of this marker is limited. Its specificity is also thought to be poor, since it is upregulated in other gynecological conditions.^(74)^ In this context, the search for novel and effective noninvasive biomarkers capable of improving endometriosis diagnosis, management and monitoring remains high on the priority list.

Circulating miRNAs, first identified as non-invasive serological markers of tumors in 2008,^(75-77)^ are promising alternative candidates. The high stability of circulating miRNAs in human plasma and their resistance to multiple sample handling procedures has been emphasized in these pioneer studies.

These same studies also established the concept of disease diagnosis based on specific cell-free miRNA signatures. Since then, miRNAs have been validated as noninvasive diagnostic markers for several diseases, including oncologic, inflammatory, cardiovascular, metabolic and reproductive disorders. miRNAs proved to be ideal diagnostic markers in oncology, as shown by differential circulating miRNA expression patterns in lung, ovarian, colorectal, prostate and breast cancer patients relative to healthy controls.^(78)^

In the female reproductive system in particular, dysregulated miRNA expression has been studied in uterine leiomyomata, in several gynecologic cancers (including adenocarcinomas), and in pregnancy disorders, such as preeclampsia and preterm birth.^(79-83)^ These small noncoding molecules associated with several diseases have been proposed as useful diagnostic candidates for endometriosis.^(84)^

In this review, miR-145 was the miRNA found to be differentially expressed in the largest number of studies (six articles). In the 46 studies analyzed, most miRNAs found to be dysregulated in endometriosis were harvested from tissue samples. Bodily fluids were seldom investigated, even though they may be used as non- or minimally invasive diagnostic tools. Also, most studies compared miRNA expression differences between the eutopic and EC of patients with endometriosis and only a few compared the endometrium of patients with endometriosis, suggesting that examinations based endometrial biopsies are difficult.

As regards dysregulated miRNAs in endometriosis patients compared in this review, 30 were found in the blood, 27 in the serum and 18 in the plasma of women with endometriosis relative to control populations. Differences in the molecular composition of serum and plasma have been well-documented.^(85,86)^

When comparing the miRNA spectrum between serum and plasma, Wang et al.,^(19)^ detected several differences in RNA levels driven by the release of certain miRNAs and other RNAs during the coagulation process, and suggested that use of plasma as the sample of choice for studying circulating miRNAs, since RNAs released during coagulation may alter the true repertoire of circulating miRNAs.

Differential expression of six miRNAs was detected in the peritoneal fluid of endometriosis patients relative to non-affected women. Hence, some miRNAs found in peritoneal fluid may play a role in the pathogenesis of endometriosis. However, given the nature of this fluid, its use is limited by the need for surgical (*i.e.*, invasive) collection.

Some points are worthy of note and should be emphasized in these studies: conflicting results. They have been reported in studies investigating miR-145, -424, -199a, -29c, -126, -16, -195 and -18a expression in the same type of sample. Major characteristics of these studies are described below.

Upregulation of miR-145 was found in the serum, in a study with 24 stages III and IV endometriosis and 24 control patients,^(51)^ and in plasma, in a study with 55 stages I and II endometriosis and 23 control patients.^(58)^ In contrast, the same miRNA was found to be downregulated in the serum in a study including 60 cases and 25 controls,^(19)^ in which disease stage was not reported.

miR-424 was downregulated in blood in a study with four patients with mild endometriosis and three controls.^(62)^ However, it was also found to be upregulated in the serum of 30 patients with minimal-mild endometriosis relative to 20 control individuals.^(52)^

miR-199a was upregulated in the serum of patients with endometriosis in two studies, one with 60 stages III and IV endometriosis and 25 control patients,^(19)^ and another with 45 endometriosis and 35 control patients.^(50)^ However, the same miRNA was found to be downregulated in the serum in a different study with 40 endometriosis and 25 control patients.^(54)^

A study with 15 clinical cases and 11 controls revealed miR-29c upregulation in the EC of women with endometriosis relative to the eutopic endometrium in the Control Group.^(39)^ This finding was further confirmed in a study including 51 women with endometriosis and 32 control women,^(25)^ in the proliferative and secretory phases of the menstrual cycle. However, conflicting results suggesting miR-29c downregulation in the EC of 20 women with endometriosis relative to the eutopic endometrium of ten control patients,^(27)^ all of them in the proliferative phase of the cycle, have been reported by a different researcher.

miR-126 was found to be upregulated in the ectopic compared to the eutopic endometrium of eight women with stages III to IV endometriosis^(45)^ in the proliferative and secretory phases of the menstrual cycle. However, miR-126 downregulation was reported in the ectopic compared to the eutopic endometrium in 31 women with stages III to IV endometriosis,^(20)^ all of them in the secretory phase of the menstrual cycle.

miR-16 and miR-195 were found to be upregulated in plasma of 33 women with endometriosis relative to 20 control patients.^(60)^ However, another study identified both downregulated in the blood of four patients with mild endometriosis relative to three controls.^(62)^

miR-18a was upregulated in serum of 24 women with stage III and IV endometriosis compared to 24 control patients.^(51)^ However, it was found to be downregulated in the blood of four patients with mild endometriosis compared to three controls.^(62)^

Conflicting results emphasize the relevance of criteria such as menstrual cycle phase, disease stage, type of sample and type of test procedure, and the need for studies with larger sample size to develop novel diagnostic tests for endometriosis.

The second objective of this review was to provide new directions for future studies aimed to identify a miRNA which may be used as a reliable biomarker and an accurate diagnostic tool for endometriosis. Sadly, according to this critical literature review no particular miRNA or miRNA combination has been validated for improved diagnosis of endometriosis to date. This may reflect the heterogeneity of the disease and resultant differences in tissue composition.^(87)^ Thus, we support the World Endometriosis Research Foundation (WERF) and Endometriosis Phenome and Biobanking Harmonization Project (EPHect) initiatives. Endometriosis research teams worldwide must join forces in order to develop large databases comprising data derived from samples obtained from patients with well-characterized endometriosis.

This is an important tool for identification and validation of biomarkers and may play a key role in biomarker investigation in future endometriosis studies.^(88)^ The inclusion of a large global pool of clinical samples collected from endometriosis patients is vital for the advancement of medical knowledge, and could be a key factor in the implementation of targeted therapies, which may enhance treatment effectiveness and improve the quality of life of endometriosis patients.

No studies investigating miRNA expression profile in the vaginal fluid were found in this literature review. This body fluid can be easily collected during gynecological examinations and, in spite of high rates of bacterial colonization, appears to be a promising source of diagnostic material.^(89,90)^ The value of differential miRNA expression in vaginal fluid as potential screening test for HPV has been examined, with interesting results.^(91-95)^

Likewise, none of the papers examined investigated miRNAs in saliva. To date, there are no scientifically proven salivary biomarkers for endometriosis. Saliva is a suitable and desirable medium for biomarker detection^(96,97)^ and its applicability to the diagnosis of endometriosis has been explored previously.^(98,99)^ Saliva is widely available and can be easily collected in a non-invasive manner, at low cost and with minimal discomfort. Therefore, it is an ideal fluid for biomarker investigation and is attracting great interest in the public health sector. The use of saliva for miRNA identification could be a potential non-invasive solution to overcome current barriers to the diagnosis of endometriosis.

This study has some limitations. When evaluating papers with contrasting results, it was not possible to tease out the factors underlying such different outcomes. Reasons explaining miRNA heterogeneity were also not found.

In this review, different studies investigating miRNA expression in endometriosis patients were discussed. Most of these studies were based on pooled or small samples. Large, well-designed clinical trials aimed to validate endometriosis-related miRNAs are needed in order to develop accurate, low-invasiveness diagnostic methods for endometriosis. The clinical impact of scientifically proven miRNA biomarkers for endometriosis will translate into better access to care and less health disparities, with potential impacts on global health. The diagnosis of endometriosis at earlier stages of the disease may lead to dramatic reduction in health costs and provide significant benefits for patients through improved health and quality of life.

## CONCLUSION

Differential miR-145 expression was reported in the largest number of studies (six articles). Most dysregulated miRNAs were harvested from tissue samples.

No particular miRNA or miRNA combination has been validated for improved diagnosis of endometriosis to date. This may have reflected the heterogeneity of the disease and resultant differences in tissue composition. Large databases comprising data derived from samples collected from patients with well-characterized endometriosis may play a key role in biomarker investigation in future studies. The use of saliva and vaginal fluid samples for miRNA identification could be a potential non-invasive solution to overcome current barriers to the diagnosis of endometriosis.
