# Supplementary material for: Overview of miRNAs for the non-invasive diagnosis of endometriosis: evidence, challenges and strategies. A systematic review
Source: Einstein (Sao Paulo). 2021 Apr 15;19:eRW5704. doi: 10.31744/einstein_journal/2021RW5704 (PMC8054530; doi:10.31744/einstein_journal/2021RW5704)
Supplement: Translation

# Translation: Visão geral de miRNAs como diagnóstico não invasivo de endometriose: evidências, desafios e estratégias. Uma revisão sistemática

**Authors:** Vitor Ulisses Monnaka, Camila Hernandes, Debora Heller, Sérgio Podgaec

**Affiliations:** 1 Brasil Faculdade Israelita de Ciências da Saúde Albert Einstein, Hospital Israelita Albert Einstein, São Paulo, SP, Brasil.; 2 Brasil Hospital Israelita Albert Einstein, São Paulo, SP, Brasil.

**Keywords:** Biomarcadores, Saliva, Soro, Fluido vaginal, Bancos de fluidos corpóreos, MicroRNAs, Endometriose/diagnóstico

## Abstract

**Objetivo:**

O objetivo do estudo foi analisar as evidências sobre miRNAs como biomarcadores para o diagnóstico de endometriose, bem como levantar informações sobre os desafios e as estratégias necessárias para tornar essas moléculas ferramentas acessíveis para uso na prática clínica.

**Métodos:**

Revisão sistemática conduzida nos bancos de dados PubMed^®^, Literatura Latino-Americana e do Caribe em Ciências da Saúde (LILACS), MEDLINE^®^ e *Web of Science* utilizando os termos de pesquisa “*endometriosis*” (todos os campos) *AND* “*miRNA*” (todos os campos), avaliando todas as publicações até maio de 2019.

**Resultados:**

A maioria dos miRNAs desregulados foram analisados a partir de amostras de tecido, o que inviabiliza seu uso como teste diagnóstico não invasivo. Todavia, 62 miRNAs foram identificados como diferencialmente expressos em amostras que poderiam ser usadas para o diagnóstico pouco invasivo de endometriose, como sangue, soro e plasma.

**Conclusão:**

Apesar de todos esses candidatos, os trabalhos são exploratórios, realizados com números pequenos de amostras, sem miRNAs específicos validados para fins diagnósticos. Estudos envolvendo principalmente amostras biológicas, visando à pesquisa translacional, deveriam ser mais explorados. O desenvolvimento de grandes bancos de dados sobre amostras, bem como o uso de saliva e fluido vaginal para identificação de miRNAs, poderia servir como recursos essenciais para as barreiras atuais no diagnóstico da endometriose.

## INTRODUÇÃO

A endometriose é uma doença comum, que afeta até 10% das mulheres em idade reprodutiva^(1,2)^ e se caracteriza pela presença de células endometriais fora da cavidade uterina. Apesar de ter sido alvo de muitos estudos, o diagnóstico da doença segue um desafio. As apresentações clínicas variam, indo desde quadros assintomáticos até com sintomatologia grave, e faltam biomarcadores diagnósticos cientificamente aprovados para o diagnóstico clínico de rotina.^(1,3)^

Exames de imagem, como a ultrassonografia pélvica e a ressonância magnética, são utilizados, principalmente em lesões profundas, porém o resultado é altamente influenciado pela experiência do examinador,^(4-7)^ o que dificulta o diagnóstico. Nos casos sem achados positivos de imagem, o diagnóstico definitivo da endometriose superficial só pode ser fechado por análise histológica da lesão em amostras geralmente coletadas por cirurgia laparoscópica.^(8,9)^ Entretanto, esse procedimento é invasivo e requer anestesia geral.

A complexidade da doença, aliada à falta de métodos diagnósticos precisos e menos invasivos, contribui para o atraso no diagnóstico, que pode levar até 11 anos.^(5,10,11)^ Consequentemente, é enorme a demanda por testes diagnósticos de alta acurácia e pouco invasivos para o diagnóstico da endometriose.^(12-16)^

Diferentes grupos de pesquisa investigaram o papel dos miRNAs (microRNA ou miR) na regulação de genes conhecidos, dada a sua associação com processos envolvidos na patogênese e na evolução dessa doença. Os miRNAs são uma classe de pequenas moléculas endógenas de RNA não codificante, envolvidas na regulação pós-transcricional da expressão gênica.^(17)^ Essas pequenas moléculas foram observadas na circulação sanguínea periférica e podem constituir biomarcadores em potencial para o diagnóstico da endometriose.^(18,19)^

A fim de determinar a real possibilidade de se empregar os miRNAs como biomarcadores para endometriose, realizamos um levantamento de todos os estudos publicados, na esperança de direcionar os passos futuros necessários para vencer os desafios associados ao uso de miRNAs na prática clínica.

## OBJETIVO

Determinar quais miRNAs têm aplicabilidade diagnóstica na endometriose e delinear os desafios e estratégias envolvidos na transformação dessas moléculas em ferramentas diagnósticas acessíveis para uso na clínica.

## MÉTODOS

A fim de identificar artigos científicos abordando associações entre endometriose e miRNA, foi realizada uma busca nas bases de dados PubMed^®^, Literatura Latino-Americana e do Caribe em Ciências da Saúde (LILACS), MEDLINE^®^ e *Web of Science*, empregando-se os unitermos *endometriosis* (todos os campos) *AND miRNA* (todos os campos).

Todas as publicações listadas até maio de 2019 (automaticamente selecionadas) foram manualmente analisadas, e somente as que envolviam padrões de expressão de miRNA validados por reação em cadeia da polimerase (PCR) em amostras clínicas de endometriose foram discutidas nesta revisão. Artigos publicados em idiomas que não o inglês, artigos baseados em cultura de células e artigos retratados, publicados em anais ou inacessíveis foram excluídos. Relatos que figuravam em mais de uma base de dados foram incluídos uma única vez no total de publicações.

Este estudo foi conduzido de acordo com a recomendação *Preferred Reporting Items for Systematic Reviews and Meta-Analyses* (PRISMA) para revisões sistemáticas, adotada pelo Hospital Israelita Albert Einstein (HIAE), localizado em São Paulo (SP). A extração de dados foi realizada em duplicata e de forma independente por dois pesquisadores, sendo, então, comparada para confirmação. Os miRNAs e seus respectivos níveis de expressão foram avaliados em amostras e populações de pacientes diferentes, assim como por ano e país de publicação.

## RESULTADOS

### Visão geral das publicações abordando miRNA e endometriose

No total, 449 artigos científicos abordando associações entre endometriose e miRNA foram encontrados nas bases de dados selecionadas para esta revisão. A maioria dos artigos (185) foi recuperada da base de dados PubMed^®^, seguida da LILACS e da MEDLINE^®^ (158) e finalmente da *Web of Science* (106). Desse conjunto de publicações, 46 se enquadraram nos critérios finais de seleção e foram selecionados para discussão nesta revisão (Figura 1).

**Figura 1:** Resultados resumidos da triagem de publicações abordando associações entre endometriose e miRNAs

SNP: polimorfismo de nucleotídeo único; LncRNAs: ácidos ribonucleicos longos não codificantes; circRNAs: ácidos ribonucleicos circulares; PCR: reação em cadeia de polimerase.

O número de publicações investigando miRNAs desregulados em mulheres com endometrioses aumentou rapidamente desde 2009, tendo aproximadamente a metade do número total de artigos (23) sido publicada nos últimos 3 anos. China e Estados Unidos foram os países com o maior número de publicações (21 e 9 artigos, respectivamente).

Nesta amostra de 46 estudos, 43 investigaram miRNAs desregulados no endométrio ectópico (EC) comparativamente ao endométrio eutópico de pacientes controle (EN), 25 foram identificados no EC comparativamente ao endométrio eutópico de mulheres com endometriose (EU), e 23 foram identificados no grupo EU comparativamente ao Grupo EN. Além disso, 27 foram identificados no soro, 18 no plasma, 30 no sangue e seis no líquido peritoneal de mulheres com endometriose em relação ao Grupo Controle. Dentre os tipos de amostras analisados, o sangue parece ser o material mais bem estudado em termos de potencial de aplicação no diagnóstico não invasivo. O resumo dos miRNAs desregulados encontrados em artigos publicados selecionados está listado em tabela 1A-1G [Table t2002] [Table t3002] [Table t4002] [Table t5002] [Table t6002] [Table t7002].

**Tabela 1A:** miRNAs diferencialmente expressos em endométrio eutópico de pacientes com endometriose comparados ao endométrio eutópico de pacientes controle

miRNA	Regulação	Endometriose n	Controle n	Referências
miR-126	RN	31	27	Liu et al.^(20)^
miR-1281	RP	38	38	Yang et al.^(21)^
miR-142-5p	RP	38	38	Yang et al.^(21)^
miR-145	RP	11	22	Zheng et al.^(22)^
miR-146a-5p	RP	38	38	Yang et al.^(21)^
miR-183-5p	RN	N/D	N/D	Shi et al.^(23)^
miR-199a	RN	12	12	Dai et al.^(24)^
miR-202-3p	RN	51	32	Braza-Boïls et al.^(25)^
miR-204	RN	38	9	Haikalis et al.^(26)^
miR-29c	RN	20	10	Long et al.^(27)^
miR-30d-5p	RP	21	25	Laudanski et al.^(28)^
miR-3152-5p	RP	21	25	Laudanski et al.^(28)^
miR-34b	RN	4	3	Burney et al.^(29)^
miR-34c-5p	RN	4	3	Burney et al.^(29)^
miR-424-5p	RN	51	32	Braza-Boils et al.^(25)^
miR-4634	RP	38	38	Yang et al.^(21)^
miR-483-5p	RN	21	25	Laudanski et al.^(30)^
miR-5187-3p	RP	21	25	Laudansk et al.^(28)^
miR-543	RN	38	38	Yang et al.^(21)^
miR-556-3p	RN	51	32	Braza-Boïls et al.^(25)^
miR-629*	RN	21	25	Laudanski et al.^(30)^
miR-9	RN	4	3	Burney et al.^(29)^
miR-940	RP	38	38	Yang et al.^(21)^

miRNA: microRNA; RN: regulação negativa; RP: regulação positiva; N/D: não disponível.

**Tabela 1B t2002:** miRNAs diferencialmente expressos em endométrio ectópico de pacientes com endometriose e endométrio eutópico de pacientes controle

miRNA	Regulação	Endometriose n	Controle n	Referências
let-7g	RP	N/D	N/D	Wright et al.^(31)^
miR-100	RP	N/D	N/D	Wright et al.^(31)^
miR-1304-3p	RP	14	10	Xu et al.^(32)^
miR-133a-3p	RP	33	17	Braicu et al.^(33)^
miR-138	RP	51	32	Braza-Boïls et al.^(25)^
miR-141	RP	22	24	Saare et al.^(34)^
miR-143	RP	11	22	Zheng et al.^(22)^
miR-145	RP	11	22	Zheng et al.^(22)^
miR-148a	RP	N/D	N/D	Wright et al.^(31)^
miR-183-5p	RN	N/D	N/D	Shi et al.^(23)^
miR-191	RP	12	12	Dong et al.^(35)^
miR-199a	RN	12	12	Dai et al.^(24)^
miR-200a	RP	22	24	Saare et al.^(34)^
miR-200b	RP	22	24	Saare et al.^(34)^
miR-200c	RN	27	12	Liang et al.^(36)^
miR-202-3p	RP	51	32	Braza-Boïls et al.^(25)^
miR-205-5p	RN	14	10	Xu et al.^(32)^
miR-20a	RP	40	20	Zhao et al.^(37)^
miR-21-3p	RP	7	7	Qi et al.^(38)^
miR-223-3p	RP	7	7	Qi et al.^(38)^
miR-29a	RP	N/D	N/D	Wright et al.^(31)^
miR-29c	RN	20	10	Long et al.^(27)^
miR-29c	RP	51	32	Braza-Boïls et al.^(25)^
miR-29c	RP	15	11	Joshi et al.^(39)^
miR-325	RP	33	17	Braicu et al.^(33)^
miR-33b	RN	20	15	Yang et al.^(40)^
miR-34c	RP	22	24	Saare et al.^(34)^
miR-3663-3p	RP	7	7	Qi et al.^(38)^
miR-3684	RP	14	10	Xu et al.^(32)^
miR-373-3p	RP	51	32	Braza-Boïls et al.^(25)^
miR-3935	RN	14	10	Xu et al.^(32)^
miR-411-5p	RP	51	32	Braza-Boïls et al.^(25)^
miR-4427	RN	14	10	Xu et al.^(32)^
miR-449a	RP	22	24	Saare et al.^(34)^
miR-450a-5p	RN	7	7	Qi et al.^(38)^
miR-451	RP	30	0	Graham et al.^(41)^
miR-4683	RP	14	10	Xu et al.^(32)^
miR-492	RP	33	17	Braicu et al.^(33)^
miR-494-5p	RP	14	10	Xu et al.^(32)^
miR-503-5p	RN	7	7	Qi et al.^(38)^
miR-520e	RP	33	17	Braicu et al.^(33)^
miR-544b	RP	14	10	Xu et al.^(32)^
miR-5481	RN	N/D	N/D	Wright et al.^(31)^
miR-652-5p	RN	14	10	Xu et al.^(32)^
miR-6747-3p	RP	14	10	Xu et al.^(32)^

miRNA: microRNA; RN: regulação negativa; N/D: não disponível; RP: regulação positiva.

**Tabela 1C t3002:** miRNAs diferencialmente expressos em endométrio ectópico e eutópico de pacientes com endometriose

miRNA	Regulação	Endometriose n	Controle n	Referências
miR-106a-5p	RN	22	0	Zhao et al.^(42)^
miR-106b-5p	RP	32	19	Yang et al.^(43)^
miR-10a	RN	38	38	Haikalis et al.^(26)^
miR-125a	RP	58	38	Ramón et al.^(44)^
miR-126	RN	31	27	Liu et al.^(20)^
miR-126	RP	8	N/D	Ohlsson Teague et al.^(45)^
miR-141	RN	8	N/D	Ohlsson Teague et al.^(45)^
miR-145	RP	8	N/D	Ohlsson Teague et al.^(45)^
miR-145-5p	RP	32	19	Yang et al.^(43)^
miR-146a-5p	RN	32	19	Yang et al.^(43)^
miR-15a-5p	RN	32	19	Yang et al.^(43)^
miR-16-5p	RP	32	19	Yang et al.^(43)^
miR-182	RN	16	N/D	Filigheddu et al.^(46)^
miR-182-5p	RN	22	0	Zhao et al.^(42)^
miR-19b-1-5p	RN	32	19	Yang et al.^(43)^
miR-200a	RN	16	N/D	Filigheddu et al.^(46)^
miR-200a-3p	RN	22	0	Zhao et al.^(42)^
miR-200b	RN	16	N/D	Filigheddu et al.^(46)^
miR-200b	RN	8	N/D	Ohlsson Teague et al.^(45)^
miR-200b	RN	32	19	Yang et al.^(43)^
miR-200c	RN	16	N/D	Filigheddu et al.^(46)^
miR-200c	RN	32	19	Yang et al.^(43)^
miR-202	RP	16	N/D	Filigheddu et al.^(46)^
miR-21	RN	38	38	Haikalis et al.^(26)^
miR-222	RP	58	38	Ramón et al.^(44)^
miR-34c	RN	22	0	Zhao et al.^(42)^
miR-424	RN	8	N/D	Ohlsson Teague et al.^(45)^
miR-424	RN	38	38	Haikalis et al.^(26)^
miR-449b	RN	51	32	Braza-Boïls et al.^(25)^
miR-449b	RN	22	0	Zhao et al.^(42)^
miR-451a	RP	41	40	Nothnick et al.^(47)^
miR-615	RP	22	0	Zhao et al.^(42)^
miR-9	RN	38	38	Haikalis et al.^(26)^
miR-99a	RP	8	N/D	Ohlsson Teague et al.^(45)^

miRNA: microRNA; RN: regulação negativa; RP: regulação positiva; N/D: não disponível.

**Tabela 1D t4002:** miRNAs diferencialmente expressos no soro de pacientes com endometriose e pacientes controle

miRNA	Regulação	Endometriose n	Controle n	Referências
let-7b	RN	24	24	Cho et al.^(48)^
let-7b-5p	RN	20	26	Nematian et al.^(49)^
miR-122	RP	60	25	Wang et al.^(19)^
miR-122	RP	45	35	Maged et al.^(50)^
miR-125b-5p	RP	24	24	Cosar et al.^(51)^
miR-125b	RP	20	26	Nematian et al.^(49)^
miR-127-3p	RN	30	20	Wang et al.^(52)^
miR-135a	RN	24	24	Cosar et al.^(51)^
miR-141	RN	60	25	Wang et al.^(19)^
miR-143-3p	RP	24	24	Cosar et al.^(51)^
miR-145	RN	60	25	Wang et al.^(19)^
miR-145-5p	RP	24	24	Cosar et al.^(51)^
miR-150-5p	RP	24	24	Cosar et al.^(51)^
miR-15b-5p	RN	30	20	Wang et al.^(52)^
miR-17	RN	80	60	Wang et al.^(53)^
miR-185-5p	RP	30	20	Wang et al.^(52)^
miR-18a-5p	RP	24	24	Cosar et al.^(51)^
miR-191	RP	12	12	Dong et al.^(35)^
miR-199a	RP	60	25	Wang et al.^(19)^
miR-199a	RP	45	35	Maged et al.^(50)^
miR-199a-5p	RN	40	25	Hsu et al.^(54)^
miR-20a-5p	RN	30	20	Wang et al.^(52)^
miR-30c-5p	RN	30	20	Wang et al.^(52)^
miR-342-3p	RP	24	24	Cosar et al.^(51)^
miR-3613-5p	RN	24	24	Cosar et al.^(51)^
miR-370	RN	20	26	Hu et al.^(55)^
miR-424-3p	RP	30	20	Wang et al.^(52)^
miR-451a	RP	41	40	Nothnick et al.^(47)^
miR-451a	RP	24	24	Cosar et al.^(51)^
miR-500a-3p	RP	24	24	Cosar et al.^(51)^
miR-542-3p	RN	60	25	Wang et al.^(19)^
miR-6755-3p	RN	24	24	Cosar et al.^(51)^
miR-9	RN	60	25	Wang et al.^(19)^
miR-99b-5p	RN	30	20	Wang et al.^(52)^

miRNA: microRNA; RN: regulação negativa; RP: regulação positiva.

**Tabela 1E t5002:** miRNAs diferencialmente expressos no plasma de pacientes com endometriose e pacientes controle

miRNA	Regulação	Endometriose n	Controle n	Referências
miR-139	RN	80	39	Nisenblat et al.^(56)^
miR-141	RN	61	65	Rekker et al.^(57)^
miR-145	RP	55	23	Bashti et al.^(58)^
miR-154-5p	RN	51	41	Pateisky et al.^(59)^
miR-155	RN	80	39	Nisenblat et al.^(56)^
miR-16	RP	33	20	Suryawanshi et al.^(60)^
miR-17-5p	RN	23	23	Jia et al.^(61)^
miR-191	RP	33	20	Suryawanshi et al.^(60)^
miR-195	RP	33	20	Suryawanshi et al.^(60)^
miR-196b	RN	51	41	Pateisky et al.^(59)^
miR-200a	RN	61	65	Rekker et al.^(57)^
miR-200b	RN	61	65	Rekker et al.^(57)^
miR-20a	RN	23	23	Jia et al.^(61)^
miR-22	RN	23	23	Jia et al.^(61)^
miR-31	RN	55	23	Bashti et al.^(58)^
miR-33a	RP	51	41	Pateisky et al.^(59)^
miR-378a	RN	51	41	Pateisky et al.^(59)^
miR-574	RN	80	39	Nisenblat et al.^(56)^

miRNA: microRNA; RN: regulação negativa; RP: regulação positiva.

**Tabela 1F t6002:** miRNAs diferencialmente expressos no sangue de pacientes com endometriose e pacientes controle

miRNA	Regulação	Endometriose n	Controle n	Referências
let-3c	RN	4	3	Azmy et al.^(62)^
let-7e	RN	4	3	Azmy et al.^(62)^
let-7f	RN	5	3	Azmy et al.^(62)^
let-7g	RN	4	3	Azmy et al.^(62)^
miR-103	RN	4	3	Azmy et al.^(62)^
miR-106b	RN	4	3	Azmy et al.^(62)^
miR-125a-5p	RN	4	3	Azmy et al.^(62)^
miR-126	RN	4	3	Azmy et al.^(62)^
miR-15b	RN	4	3	Azmy et al.^(62)^
miR-16	RN	4	3	Azmy et al.^(62)^
miR-17	RN	4	3	Azmy et al.^(62)^
miR-181b	RN	4	3	Azmy et al.^(62)^
miR-18a	RN	4	3	Azmy et al.^(62)^
miR-194	RN	4	3	Azmy et al.^(62)^
miR-195	RN	4	3	Azmy et al.^(62)^
miR-19a	RN	4	3	Azmy et al.^(62)^
miR-19b	RN	4	3	Azmy et al.^(62)^
miR-20a	RN	4	3	Azmy et al.^(62)^
miR-21	RN	4	3	Azmy et al.^(62)^
miR-22	RN	4	3	Azmy et al.^(62)^
miR-26a	RN	4	3	Azmy et al.^(62)^
miR-26b	RN	4	3	Azmy et al.^(62)^
miR-27a	RN	4	3	Azmy et al.^(62)^
miR-27b	RN	4	3	Azmy et al.^(62)^
miR-30a	RN	4	3	Azmy et al.^(62)^
miR-374a	RN	4	3	Azmy et al.^(62)^
miR-374b	RN	4	3	Azmy et al.^(62)^
miR-424	RN	4	3	Azmy et al.^(62)^
miR-7	RN	4	3	Azmy et al.^(62)^
miR-93	RN	4	3	Azmy et al.^(62)^

miRNA: microRNA; RN: regulação negativa.

**Tabela 1G t7002:** miRNAs diferencialmente expressos no líquido peritoneal de pacientes com endometriose e pacientes controle

miRNA	Regulação	Endometriose n	Controle n	Referências
miR-106b-3p	RP	126	45	Marí-Alexandre et al.^(63)^
miR-122	RP	45	35	Maged et al.^(50)^
miR-130b	RP	6	3	Chen et al.^(64)^
miR-199a	RP	45	35	Maged et al.^(50)^
miR-451a	RP	126	45	Marí-Alexandre et al.^(63)^
miR-486-5p	RP	126	45	Marí-Alexandre et al.^(63)^

miRNA: microRNA; RP: regulação positiva.

Ao todo, 33 miRNAs foram analisados em mais de um estudo. Destes, 13 foram analisados em amostras do mesmo tipo. Os miRNAs identificados em mais de um estudo e fluido corporal encontram-se descritos na tabela 2.

**Tabela 2:** Resumo dos miRNAs desregulados identificados em mais de um estudo em diferentes amostras

Total	EU *versus* EN	EC *versus* EN	EC *versus* EU	Plasma	Soro	Sangue	LP	Referências
6	miR-145	miR-145	miR-145	miR-145	miR-145			Wang et al.,^(19)^ Zheng et al.,^(22)^ Yang et al.,^(43)^ Ohlsson Teague et al.,^(45)^ Cosar et al.^(51)^ e Bashti et al.^(58)^
5		miR-200b	miR-200b	miR-200b				Saare et al.,^(34)^ Yang et al.,^(43)^ Ohlsson Teague et al.,^(45)^ Filigheddu et al.^(46)^ e Rekker et al.^(57)^
5	miR-424		miR-424		miR-424	miR-424		Braza-Boils et al.,^(25)^ Haikalis et al.,^(26)^ Ohlsson Teague et al.,^(45)^ Wang et al.^(52)^ e Azmy et al.^(62)^
4	miR-199a	miR-199a			miR-199a		miR-199a	Wang et al.,^(19)^ Dai et al.,^(24)^ Maged et al.^(50)^ e Hsu et al.^(54)^
4		miR-141	miR-141	miR-141	miR-141			Wang et al.,^(19)^ Saare et al.,^(34)^ Ohlsson Teague et al.^(45)^ e Rekker et al.^(57)^
4		miR-20a		miR-20a	miR-20a	miR-20a		Zhao et al.,^(37)^ Wang et al.,^(52)^ Jia et al.^(61)^ e Azmy et al.^(62)^
4		miR-200a	miR-200a	miR-200a				Saare et al.,^(34)^ Zhao et al.,^(42)^ Filigheddu et al.^(46)^ e Rekker et al.^(57)^
3	miR-29c	miR-29c	miR-29c					Braza-Boils et al.,^(25)^ Long et al.^(27)^ e Joshi et al.^(39)^
3	miR-34c	miR-34c	miR-34c					Braza-Boïls et al.,^(25)^ Saare et al.^(34)^ e Joshi et al.^(39)^
3		miR-200c	miR-200c					Liang et al.,^(36)^ Yang et al.^(43)^ e Filigheddu et al.^(46)^
3		miR-21	miR-21			miR-21		Haikalis et al.,^(26)^ Qi et al.^(38)^ e Azmy et al.^(62)^
3	miR-126		miR-126			miR-126		Liu et al.,^(20)^ Ohlsson Teague et al.^(45)^ e Azmy et al.^(62)^
3			miR-16	miR-16		miR-16		Yang et al.,^(43)^ Suryawanshi et al.^(60)^ e Azmy et al.^(62)^
3			miR-451a		miR-451a		miR-451a	Nothnick et al.,^(47)^ Cosar et al.^(51)^ e Marí-Alexandre et al.^(63)^
3	miR-9		miR-9		miR-9			Wang et al.,^(19)^ Haikalis et al.^(26)^ e Burney et al.^(29)^
3			miR-106b			miR-106b	miR-106b	Yang et al.,^(43)^ Azmy et al.^(62)^ e Marí-Alexandre et al.^(63)^
3				miR-17	miR-17	miR-17		Wang et al.,^(53)^ Jia et al.^(61)^ e Azmy et al.^(62)^
2					miR-122		miR-122	Wang et al.^(19)^ e Maged et al.^(50)^
2			miR-449b					Braza-Boïls et al.^(25)^ e Zhao et al.^(42)^
2		miR-191		miR-191	miR-191			Dong et al.^(35)^ e Suryawanshi et al.^(60)^
2	miR-202	miR-202	miR-202					Braza-Boïls et al.^(25)^ e Filigheddu et al.^(46)^
2		miR-143			miR-143			Zheng et al.^(22)^ e Cosar et al.^(51)^
2				miR-22		miR-22		Jia et al.^(61)^ e Azmy et al.^(62)^
2		miR let-7g				miR let-7g		Wright et al.^(31)^ e Azmy et al.^(62)^
2					miR-15b	miR-15b		Wanget al.^(52)^ e Azmy et al.^(62)^
2			miR-125a			miR-125a		Ramón et al.^(44)^ e Azmy et al.^(62)^
2				miR-195		miR-195		Suryawanshi et al.^(60)^ e Azmy et al.^(62)^
2					miR-18a	miR-18a		Cosar et al.^(51)^ e Azmy et al.^(62)^
2			miR-19b			miR-19b		Yanget al.^(43)^ e Azmy et al.^(62)^
2	miR-146a		miR-146a					Yang et al.^(21)^ e Yang et al.^(43)^
2			miR-182					Zhao et al.^(42)^ e Filigheddu et al.^(46)^
2					miR-125b			Nematian et al.^(49)^ e Cosar et al.^(51)^
2					miR-let-7b			Cho et al.^(48)^ e Nematian et al.^(49)^

EU: endométrio eutópico de mulheres com endometriose; EN: endométrio eutópico de pacientes controle; EC: endométrio ectópico; LP: líquido peritoneal.

Dentre os 62 miRNAs identificados em amostras com possível aplicabilidade no diagnóstico pouco invasivo da endometriose, como sangue, soro e plasma, 20 também se mostraram desregulados em outros tipos de tecidos, tais EC e eutópico, e no líquido peritoneal. Destes, 35% foram identificados no mesmo tipo de tecido em mais de um estudo, incluindo miR-200b, miR-145, miR-199a, miR-424, miR-200a, miR-126 e miR-451a. Treze miRNAs mostraram-se desregulados de forma positiva ou negativa, a saber: miR-125b, miR-let-7b, miR-122, miR-451a e miR-199a no soro; miR-29c no EC em relação ao Grupo EN; e miR-145, miR-200b, miR-424, miR-200a, miR-200c, miR-449b e miR-182 no EC em relação ao EC de mulheres com endometriose (Tabela 3).

**Tabela 3:** Caracterização da expressão de miRNAs para regulação positiva e negativa em diferentes amostras

miRNA	n	EU *versus* EN		EC *versus* EN		EC *versus* EU		Plasma	Soro	Sangue	LP
		EU	EN	EC	EN	EC	EU				
miR-145	6	↑ (1)	↓ (1)	↑ (1)	↓ (1)	↑ (2)	↓ (2)	↑ (1)	↑ (1) /↓ (1)		
miR-200b	5			↑ (1)	↓ (1)	↓ (3)	↑ (3)	↓ (1)			
miR-424	5	↓ (1)	↑ (1)			↓ (2)	↑ (2)		↑ (1)	↓ (1)	
miR-199a	4	↓ (1)	↑ (1)	↓ (1)	↑ (1)			↑ (1)	↑ (2)/↓ (1)		↑ (1)
miR-141	4			↑ (1)	↓ (1)	↓ (1)	↑ (1)	↓ (1)	↓ (1)		
miR-20a	4			↑ (1)	↓ (1)			↓ (1)	↓ (1)	↓ (1)	
miR-200a	4			↑ (1)	↓ (1)	↓ (2)	↑ (2)	↓ (1)			
miR-29c	3	↓ (1)	↑ (1)	↑ (2) /↓ (1)	↓ (2) / ↑(1)	↓ (1)	↑ (1)				
miR-34c	3	↓ (1)	↑ (1)	↑ (1)	↓ (1)	↓ (1)	↑ (1)				
miR-200c	3			↓ (1)	↑ (1)	↓ (2)	↑ (2)				
miR-21	3			↑ (1)	↓ (1)	↓ (1)	↑ (1)			↓ (1)	
miR-126	3	↓ (1)	↑ (1)			↑ (1)/↓ (1)	↓ (1)/↑ (1)			↓ (1)	
miR-16	3					↑ (1)	↓ (1)	↑ (1)		↓ (1)	
miR-451a	3					↑ (1)	↓ (1)		↑ (2)		↑ (1)
miR-9	3	↓ (1)	↑ (1)			↓ (1)	↑ (1)		↓ (1)		
miR-106b	3					↑ (1)	↓ (1)			↓ (1)	↑ (1)
miR-17	3							↓ (1)	↓ (1)	↓ (1)	
miR-122	2								↑ (2)		↑ (1)
miR-449b	2					↓ (2)	↑ (2)				
miR-191	2			↑ (1)	↓ (1)			↑ (1)	↑ (1)		
miR-202	2	↓ (1)	↑ (1)	↑ (1)	↓ (1)	↑ (1)	↓ (1)				
miR-143	2			↑ (1)	↓ (1)				↑ (1)		
miR-22	2							↓ (1)		↓ (1)	
miR let-7g	2			↑ (1)	↓ (1)					↓ (1)	
miR-15b	2								↓ (1)	↓ (1)	
miR-125a	2					↑ (1)	↓ (1)			↓ (1)	
miR-195	2							↑ (1)		↓ (1)	
miR-18a	2								↑ (1)	↓ (1)	
miR-19b	2					↓ (1)	↑ (1)			↓ (1)	
miR-146a	2	↑ (1)	↓ (1)			↓ (1)	↑ (1)				
miR-182	2					↓ (2)	↑ (2)				
miR-125b	2								↑ (2)		
miR-let-7b	2								↓ (2)		

↑ regulação positiva; ↓ regulação negativa.

miRNA: microRNA; EU: endométrio eutópico de mulheres com endometriose; EN: endométrio eutópico de pacientes controle; EC: endométrio ectópico; PF: líquido peritoneal.

## DISCUSSÃO

A endometriose pode ser uma doença debilitante e causa de má qualidade de vida.^(65)^ Essa enfermidade tem relação com dismenorreia, dispareunia profunda, dor pélvica crônica e infertilidade,^(66,67)^ sendo considerada um problema de saúde pública, devido ao impacto sobre a saúde física e psicológica das pacientes e ao impacto socioeconômico decorrente dos custos do diagnóstico, do tratamento e do controle clínico.^(68)^

Atualmente, o diagnóstico definitivo da endometriose se baseia na análise histológica da lesão, geralmente empregando-se amostras obtidas por cirurgia laparoscópica.^(69)^ Entretanto, os métodos de imagem constituem alternativas diagnósticas não invasivas importantes para a endometriose ovariana e profunda. Tanto a abordagem cirúrgica como a não cirúrgica requerem habilidade professional considerável e disponibilidade de dados específicos, o que pode representar enorme carga econômica e de saúde em países em desenvolvimento.^(4-9)^

Nas últimas três décadas, pesquisadores do mundo todo tentaram identificar testes não invasivos capazes de reduzir o tempo necessário para o diagnóstico da endometriose. O CA-125 pode ser detectado no sangue ou no líquido peritoneal e é um dos biomarcadores mais bem estudados. Em alguns estudos de casos, a dosagem de CA-125 mostrou-se promissora, principalmente no diagnóstico da endometriose mais invasiva, desde que realizada no início do ciclo menstrual.^(70-72)^

Apesar dos resultados conflitantes referentes ao valor do CA-125 enquanto biomarcador decisivo e importante em revisões recentes, segundo Socolov et al., o CA-125 continua sendo o mais indicado para o diagnóstico e o monitoramento da endometriose.^(73)^ Em uma revisão Cochrane mais recente, publicada em 2016, Nisenblat et al., compararam a acurácia de combinações de testes não invasivos para diagnóstico da endometriose pélvica com o diagnóstico cirúrgico, empregando como padrão de referência ensaios randomizados controlados ou estudos transversais publicados até meados de 2015. Os autores concluíram que nenhum dos biomarcadores investigados (incluindo o CA-125) pode ser devidamente avaliado por falta de evidência suficiente ou de boa qualidade, dada a heterogeneidade e o alto risco de viés nos estudos selecionados.^(15)^

Por mostrar-se mais elevado nos estágios avançados da endometriose, a sensibilidade do CA-125 é limitada. Além disso, a especificidade desse marcador é baixa, devido à sua regulação positiva em outras patologias ginecológicas.^(74)^ Nesse contexto, a busca por novos biomarcadores eficazes e não invasivos, capazes de aprimorar o diagnóstico, o tratamento e o monitoramento da endometriose, permanece no topo da lista de prioridades.

Dentre os candidatos promissores, destacam-se os miRNAs circulantes, identificados pela primeira vez como marcadores sorológicos não invasivos para tumores em 2008.^(75-77)^ A alta estabilidade dos miRNAs circulantes no plasma humano e sua resistência a diversos procedimentos de manipulação de amostras foram enfatizadas nesses estudos pioneiros.

Além disso, esses mesmos estudos estabeleceram o conceito de diagnóstico baseado em assinaturas de miRNAs livres de células específicas. Desde então, os miRNAs foram validados como marcadores diagnósticos não invasivos para diversas doenças, incluindo distúrbios oncológicos, inflamatórios, cardiovasculares, metabólicos e reprodutivos. Os miRNAs se revelaram marcadores ideais na oncologia, conforme demonstram padrões diferenciais de expressão de miRNAs circulantes em pacientes com câncer pulmonar, ovariano, colorretal, prostático e de mama, em relação a controles saudáveis.^(78)^

No sistema reprodutivo feminino em particular, a expressão desregulada de miRNAs foi estudada em fibroides uterinos, em diversos cânceres ginecológicos (incluindo adenocarcinomas) e em distúrbios de parto, como a pré-eclâmpsia e o parto prematuro.^(79-83)^ Essas pequenas moléculas não codificantes associadas a diversas doenças figuram entre os candidatos úteis para o diagnóstico da endometriose.^(84)^

Nesta revisão, o miRNA com expressão diferencial no maior número de estudos foi o miR-145 (seis artigos). Nos 46 estudos analisados, a maioria dos miRNAs que se mostraram desregulados na endometriose foi coletada de amostras teciduais. Portanto, os fluidos corpóreos foram pouco investigados, embora possam ser úteis enquanto ferramentas diagnósticas não ou pouco invasivas. Além disso, a maioria dos estudos comparou as diferenças de expressão de miRNAs entre o endométrio eutópico e o EC de pacientes com endometriose, sendo poucos os que estabeleceram comparações com o endométrio de pacientes com endometriose, ressaltando a dificuldade dos exames baseados em biópsias endometriais.

Dentre os miRNAs desregulados em pacientes com endometriose comparados nesta revisão, 30 foram detectados no sangue, 27 no soro e 18 no plasma de mulheres com endometriose comparadas com populações controle. As diferenças de composição molecular entre o soro e o plasma são bem conhecidas.^(85,86)^

Ao comparar o espectro de miRNAs no soro e no sangue, Wang et al.,^(19)^ observaram várias discrepâncias nas concentrações de RNAs determinadas pela liberação de certos miRNAs e outros RNAs durante a coagulação e sugeriram que o plasma seja utilizado como amostra de eleição para estudo de miRNAs circulantes, uma vez que os RNAs liberados durante a coagulação podem alterar o repertório real de miRNAs circulantes.

A expressão diferencial de seis miRNAs foi identificada no líquido peritoneal de pacientes com endometriose em relação a mulheres não afetadas. Portanto, alguns miRNAs presentes no líquido peritoneal podem atuar na patogênese da endometriose. Entretanto, dada a natureza desse fluido, seu emprego é limitado pela necessidade de coleta cirúrgica, ou seja, invasiva.

Um ponto digno de nota nesses estudos foram os resultados conflitantes. Eles foram relatados em estudos que investigaram a expressão dos miR-145, -424, -199a, -29c, -126, -16, -195 e -18a no mesmo tipo de amostra. As principais características desses estudos encontram-se descritas a seguir.

A regulação positiva do miR-145 foi observada no soro, em estudo com 24 pacientes com endometriose estágios III e IV e 24 pacientes controle,^(51)^ e no plasma em um estudo com 55 pacientes com endometriose estágios I e II e 23 pacientes controle.^(58)^ Em contrapartida, estudo com 60 casos (estágio da doença não relatado) e 25 controles relatou regulação negativa desse mesmo miRNA no soro.^(19)^

Um estudo com quatro pacientes com endometriose leve e três controles relatou regulação negativa do miR-424 no sangue.^(62)^ Entretanto, esse mesmo marcador mostrou-se regulado de forma positiva no soro de 30 pacientes com endometriose mínima leve em relação a 20 indivíduos controle.^(52)^

A regulação positiva do miR-199a foi detectada no soro de pacientes com endometrioses em dois estudos, sendo um com 60 pacientes com endometriose estágios III e IV e 25 pacientes controle^(19)^ e o outro com 45 pacientes com endometriose e 35 pacientes controle.^(50)^ Entretanto, a regulação negativa do mesmo miRNA no soro foi descrita em outro estudo envolvendo 40 pacientes com endometriose e 25 pacientes controle.^(54)^

Um estudo com 15 casos clínicos e 11 controles revelou regulação positiva do miR-29c no EC de mulheres com endometriose em relação ao endométrio eutópico do Grupo Controle.^(39)^ Esse achado foi reconfirmado em outro estudo envolvendo 51 mulheres com endometriose e 32 controle^(25)^ nas fases proliferativa e secretória do ciclo menstrual. Entretanto, resultados conflitantes, sugerindo regulação negativa do miR-29c no EC de 20 mulheres com endometriose em relação ao endométrio eutópico de dez pacientes controle,^(27)^ todas exclusivamente na fase proliferativa do ciclo, foram relatados por outro pesquisador.

A regulação positiva do miR-126 no EC comparativamente ao endométrio eutópico foi detectada em oito mulheres com endometriose estágios III e IV^(45)^ nas fases proliferativa e secretória do ciclo menstrual. Entretanto, a regulação negativa do miR-126 foi relatada no EC comparativamente ao endométrio eutópico em 31 mulheres com endometriose estágios III e IV,^(20)^ todas na fase secretória do ciclo menstrual.

A regulação positiva do miR-16 e do miR-195 foi observada no plasma de 33 mulheres com endometriose comparadas com 20 pacientes controles.^(60)^ Entretanto, outro estudo identificou regulação negativa de ambos no sangue de quatro pacientes com endometriose leve comparadas com três controles.^(62)^

A regulação positiva do miR-18a foi observada no soro de 24 mulheres com endometriose estágios III e IV em relação a 24 pacientes controle.^(51)^ Entretanto, a regulação negativa foi identificada no sangue de quatro pacientes com endometriose leve em relação a três controles.^(62)^

A discrepância nos resultados destaca a relevância de critérios como fase do ciclo menstrual, estágio da doença, tipo de amostra e tipo de procedimento, bem como a necessidade de estudos com maior número de pacientes para o desenvolvimento de testes diagnósticos para a endometriose.

O segundo objetivo deste estudo foi nortear estudos futuros destinados à identificação de um miRNA que possa constituir um biomarcador confiável e uma ferramenta para o diagnóstico preciso da endometriose. De acordo com essa revisão crítica da literatura, infelizmente não há um miRNA específico ou uma combinação de miRNAs devidamente validada para melhorar o diagnóstico da endometriose. Isso pode refletir a heterogeneidade da doença e, consequentemente, as diferenças de composição tecidual.^(87)^ Por esse motivo, apoiamos as iniciativas da *World Endometriosis Research Foundation* (WERF) e do *Endometriosis Phenome and Biobanking Harmonization Project* (EPHect). O esforço conjunto de grupos mundiais de pesquisa para a criação de grandes bancos de dados derivados de amostras coletadas de pacientes com endometriose bem caracterizada é fundamental.

Essa é uma importante ferramenta para a identificação e a validação de biomarcadores, que pode desempenhar papel fundamental na investigação de biomarcadores em estudos futuros.^(88)^ A inclusão de um *pool* global de amostras clínicas coletadas de pacientes com endometriose é fundamental para o avanço do conhecimento médico e pode ser de grande valia na implementação de terapias-alvo, melhorando a efetividade do tratamento e a qualidade de vida das pacientes que sofrem de endometriose.

Nesta revisão de literatura, não foram encontrados estudos investigando o perfil de expressão de miRNAs no fluido vaginal. Esse fluido corporal pode ser facilmente coletado durante o exame ginecológico e, apesar da alta taxa de colonização bacteriana, parece ser fonte promissora de material diagnóstico.^(89,90)^ O valor da expressão diferencial de miRNAs no fluido vaginal enquanto teste potencial de rastreio de HPV foi avaliado, com resultados interessantes.^(91-95)^

Da mesma forma, nenhum dos artigos aqui revisados investigou miRNAs na saliva. Até o momento, não existem biomarcadores salivares para endometriose cientificamente comprovados. O emprego da saliva como meio de detecção de biomarcadores é adequado e desejável^(96,97)^ e foi previamente explorado no diagnóstico da endometriose.^(98,99)^ A ampla disponibilidade, aliada à facilidade de coleta por método não invasivo, com baixo custo e desconforto mínimo, faz da saliva um material ideal para a pesquisa de biomarcadores, que vem despertando grande interesse na esfera da saúde pública. O uso da saliva para identificação de miRNAs seria uma solução não invasiva em potencial para a eliminação dos obstáculos vigentes para o diagnóstico da endometriose.

Este estudo tem algumas limitações. Na avaliação de artigos com resultados conflitantes, não foi possível identificar os fatores responsáveis pelos diferentes desfechos. As razões por trás da heterogeneidade de miRNA também não foram encontradas.

Diversos estudos que investigaram a expressão de miRNAs em pacientes com endometriose foram discutidos nesta revisão. Em sua maioria, esses estudos foram baseados em amostras agrupadas ou pequenas. Ensaios clínicos grandes e bem planejados, destinados à validação de miRNAs associados à endometriose, fazem-se necessários para o desenvolvimento de métodos diagnósticos acurados e pouco invasivos. O impacto clínico do emprego dos miRNAs cientificamente comprovados como biomarcadores para endometriose se traduzirá em mais amplo acesso a cuidados e menor disparidade em saúde, com potencial impacto sobre a saúde global. O diagnóstico da doença em estágios mais precoces pode se traduzir em uma redução dramática dos custos de saúde, além de beneficiar os pacientes em termos de melhor saúde e qualidade de vida.

## CONCLUSÃO

A expressão diferencial do miR-145 foi relatada no maior número de estudos (seis artigos). Em sua maioria, os miRNAs desregulados foram extraídos de amostras teciduais.

Nenhum miRNA específico ou combinação de miRNAs foi validado para melhorar o diagnóstico da endometriose, o que pode refletir a heterogeneidade da doença e, consequentemente, diferenças de composição tecidual. Bancos de dados grandes, que contenham dados derivados de amostras coletadas de pacientes com endometriose bem caracterizada, podem ter papel fundamental na investigação de biomarcadores em estudos futuros. O uso de amostras de saliva e fluido vaginal para a identificação de miRNAs poderia representar uma solução não invasiva em potencial para a eliminação das barreiras vigentes para diagnóstico da endometriose.
